# Sensitivity of forces to wall transpiration in flow past an aerofoil

**DOI:** 10.1098/rspa.2015.0618

**Published:** 2015-12-08

**Authors:** X. Mao

**Affiliations:** School of Engineering and Computing Sciences, Durham University, Durham DH1 3LE, UK

**Keywords:** sensitivity, wall transpiration, flow separation

## Abstract

The adjoint-based sensitivity analyses well explored in hydrodynamic stability studies are extended to calculate the sensitivity of forces acting on an aerofoil with respect to wall transpiration. The magnitude of the sensitivity quantifies the controllability of the force, and the distribution of the sensitivity represents a most effective control when the control magnitude is small enough. Since the sensitivity to streamwise control is one order smaller than that to the surface-normal one, the work is concentrated on the normal control. In direct numerical simulations of flow around a NACA0024 aerofoil, the unsteady controls are far less effective than the steady control owing to the lock-in effect. At a momentum coefficient of 0.0008 and a maximum control velocity of 3.6% of the free-stream velocity, the steady surface-normal control reduces drag by 20% or enhances lift by up to 140% at *Re*=1000. A suction around the low-pressure region on the upper surface upstream of the separation point is found to reduce drag and enhance lift. At higher Reynolds numbers, the uncontrolled flow becomes three dimensional and the sensitivity diverges owing to the chaotic dynamics of the flow. Then the mechanism identified at lower Reynolds numbers is exploited to obtain the control, which is localized and can be generated by a limited number of actuators. The control to reduce drag or enhance lift is found to suppress unsteadiness, e.g. vortex shedding and three-dimensional developments. For example, at *Re*=2000 and *α*=10°, the control with a momentum coefficient of 0.0001 reduces drag by 20%, enhances lift by up to 200% and leads to a steady controlled flow.

## 1. Introduction

Flow over aircraft wings or turbine blades at a high angle of attack is commonly associated with boundary-layer separations and unsteady vortex shedding, which would cause vortex-induced vibration, potential damage of structures and a serious increase of the mean drag and the lift fluctuation. Numerous flow control strategies have been proposed to control the stability of the boundary layers, vortex shedding in the wake, or the drag and lift forces acting on the body, by means of either active or passive control [1]. Active control, which involves energy input, is commonly achieved by generating non-zero velocity on the surface of a solid body, while other active control techniques exist, such as using near-wall forcing to reduce turbulence drag [2], generating travelling waves on the rear surface of a cylinder to suppress vortex shedding [3], and exciting electrodes to generate plasma sheets and modify the near wake of a cylinder [4]. In the rest of this section, the wall-normal transpiration control, which is also the focus of the current work, is reviewed in §1a, and the sensitivity analyses used to calculate the control are introduced in §1b.

### (a) Literature review of the wall-normal control

The wall-normal control, i.e. blowing or suction normal to the surface, can be categorized as steady, low-frequency, high-frequency and time-dependent but non-periodic controls depending on the frequency of the wall forcing. The steady control refers to steady blowing/suction, while the low- and high-frequency controls refer to periodic oscillations of the wall-normal velocity component which can be generated by synthetic jets [5]. These three types of normal control are open-loop control techniques, while the last one, i.e. the time-dependent but non-periodic control, is often associated with feedback control. Since the control addressed in this work is based on fixed control objectives and can be regarded as an open-loop control, only the first three types of normal control are reviewed in the following.

Steady normal control introduced from the base of a solid body has been widely used to control wake stabilities and vortex shedding [6,7]. For the control of forces, Delaunay & Kaiktsis [8] used base blowing to control the flow around a circular cylinder. They achieved a 14% reduction of drag at a maximum control velocity of approximately 0.37 (normalized by the free-stream velocity as will be used in the following) at Reynolds number *Re*=90 and observed that most of the reduction is attributed to the pressure component. Kametani & Fukagata [9] found that uniform blowing (or suction) with a relative control velocity of 0.01 reduces (or increases) friction drag in a boundary-layer flow at *Re*=3000. Kim & Choi [10] applied steady blowing/suction varying sinusoidally in the spanwise direction on the upper and lower surfaces of a cylinder and observed that a symmetric (‘in-phase’) control forcing reduces drag significantly and also suppresses vortex shedding more efficiently than base bleeding. They achieved about 20% drag reduction at a maximum relative control velocity 0.1 at *Re*=100. The mechanism of this control is attributed to the phase mismatch in the spanwise direction, similar to effects induced by spanwise geometry variation [11,12].

The low-frequency normal control refers to periodic blowing and suction at frequencies commensurate with natural frequencies of the flow. Wu *et al.* [13] discussed two natural frequencies, i.e. shear layer frequency and vortex shedding frequency, in flow around a NACA0012 aerofoil at a large angle of attack (20°≤*α*≤30°). They achieved a 70% lift enhancement accompanied by an over 20% increase of drag at a maximum relative control velocity of 0.42 and control frequencies around the latter. Raju *et al.* [14] identified three natural frequencies corresponding to the shear layer, separation bubble and wake regions in two-dimensional simulations to control separation in flow around a NACA4418 aerofoil at *Re*=40 000 and *α*=18°. They suggested that the control around the separation bubble frequency, which would be close to the vortex shedding frequency at a large angle of attack as presented by Wu *et al.* [13], is the most effective to diminish separation. Imposing control at this optimal frequency, they observed a 1% increase of lift and a 39% reduction of drag at a maximum relative control velocity 0.1 and a momentum coefficient (relative momentum of the control with respect to the free stream) of 1.2×10^−4^−1.9×10^−4^. A similar result was observed in another study of flow around a thick elliptic plate [15]. For controls at shear layer frequencies, Hong [16] used synthetic jets with frequencies in the lower range of Tollmien–Schlichting waves to activate boundary-layer instabilities so as to prevent laminar separation and accelerate laminar–turbulence transition.

The high-frequency normal control refers to control at frequencies one order larger than natural frequencies of the flow. Therefore, the control effects are decoupled from instabilities of the uncontrolled flow. At such high frequencies, the control generates localized concentrations of ‘trapped’ vorticity, and subsequently alters the shape of the surface as passive control devices [17]. Jeon *et al.* [18] observed nearly 50% drag reduction in flow around a sphere at *Re*=10^5^ under the control of synthetic jets with an optimal frequency around 16 times the uncontrolled vortex shedding frequency and a maximum relative control velocity 0.1. The mechanism of drag reduction is attributed to the delay of the main separation owing to the formation of a bubble and reattachment upstream of the main separation. This control mechanism can be considered as altering the body surface; a similar control effect was achieved by placing a protuberance with various shapes upstream of the separation points in a cylinder flow [19]. Unlike the steady and low-frequency controls, the mechanism of high-frequency control associated with trapped vortices is nonlinear.

The majority of the control forcing discussed above is generated by localized control actuators, with a few exceptions, e.g. optimally distributed control across the entire body surface or rigid rotation of a circular solid body. In this work, the control is calculated by scaling the sensitivity of forces acting on a solid body with respect to surface forcing. This control is optimal in the linear sense (the magnitude of the control velocity is small enough) and therefore the nonlinear control mechanisms associated with the generation of trapped vortices in the high-frequency normal control will not be addressed. Since the sensitivity is distributed across the whole surface, the control is continuous instead of concentrated on a limited number of segments of the body surface. It is noted that, even though the exact form of this control may not be physically realizable, the study highlights localized regions where discrete control strategies can be developed, as presented in §4f.

### (b) Introduction of sensitivity analyses

Most sensitivity studies in fluid dynamics have targeted the sensitivity of kinetic energy with respect to external forcing, which is modelled as source terms of the linearized Navier–Stokes (NS) equation [20–22]. The calculated optimal external forcing leads to maximum energy growth and therefore facilitates understanding of fluid physics, e.g. stabilities or non-normal noise amplifications. Similar studies have been conducted to calculate the optimal initial perturbation, which is the initial condition of the linearized NS equation and induces the largest energy growth over a given time horizon [23]. For boundary conditions, the methodology to calculate optimal (most energetic) Dirichlet-type boundary conditions has been established and used to compute optimal inflow conditions in stenotic flow and vortex flow [24,25]. It is noted that most of the studies of optimal perturbations, including initial perturbations, external forcing and boundary perturbations, addressed in the literature, have targeted perturbation energy growth and the term ‘optimal’ is synonymous with ‘most energetic’. This most energetic study has been recently extended to investigate the sensitivity of forces or flow separations to external forcing [26,27].

Among the three types of perturbations, i.e. external forcing, initial and boundary perturbations, the last one is an intuitive option for active flow control. Most of the physical controls are introduced on the solid surface and are therefore in the form of boundary perturbations to the uncontrolled flow; external forcing can be generated by extra small cylinders representing a passive control [21,27]; initial conditions can be convected out of the domain after a finite time interval and therefore cannot be used as a sustainable and reliable control.

The sensitivity of forces acting on a solid body with respect to the boundary perturbation can be scaled as a control, which is most effective to modify the forces in the linear sense. The methodology to calculate this sensitivity or control can be derived from that to compute the most energetic boundary velocity, by modifying the objective function from the (boundary perturbation) induced energy to induced forces. The calculation of the optimal control involves a single integration of an adjoint equation, which has been extensively used in hydrodynamic studies [23] and optimization of geometry or control parameters [28]. The adjoint equation used in this work is two dimensional and its computational cost is close to the two-dimensional linearized NS equation. Owing to the linear assumptions of this sensitivity study, there are no iterative calls on the adjoint equation. Therefore, the computational cost of the sensitivity or the control is close to a two-dimensional direct numerical simulation (DNS).

In this work, an aerofoil flow is adopted as an example to illustrate the sensitivity and the associated control. In the following, the methodology to calculate the sensitivity and the control is introduced in §2; the numerical method, discretization and convergence are discussed in §3; the sensitivity and nonlinear control effects of the control are presented in §4; finally, the conclusion is drawn in §5.

## 2. Methodology

### (a) Governing equations

The controlled flow can be decomposed as the sum of an uncontrolled flow, which is synonymous with base flow in hydrodynamic stability and other perturbation studies, and a control-induced flow, i.e. $\left(\hat{\text{ u}},\hat{p})=(\text{ U},P)+(\text{ u},p\right)$, where $\hat{\text{ u}}$, ***U*** and ***u*** are total, uncontrolled and control-induced velocity terms, respectively, and $\hat{p}$, *P* and *p* are total, uncontrolled and control-induced pressure terms, respectively.

The total controlled flow $\left(\hat{\text{ u}},\hat{p}\right)$ and the uncontrolled flow (***U***,*P*) satisfy the NS equation. If the control is small enough, so does the control-induced flow. Then the control-induced velocity and pressure can be governed by the linearized NS equation,

$$\partial_{t}\text{u}+\text{U}\cdot \nabla \text{u}+\text{u}\cdot \nabla \text{U}+\nabla p-Re^{-1}\nabla^{2}\text{u}=0,\;\text{with}\;\nabla \;\cdot \;\text{u}=0,$$

or more compactly as

2.1 $$\partial_{t}\text{u}-L(\text{u})=0,$$

where L is a linearized operator. The initial condition of ***u*** is set to zero, corresponding to a zero control-induced flow at the beginning of the simulation. For boundary conditions, on the inflow and far-field boundaries, zero Dirichlet and computed Neumann conditions are adopted for velocity and pressure, respectively [29]; on the outflow boundary, a zero Neumann velocity condition normal to the boundary and zero Dirichlet pressure condition are implemented; on the controlled boundary referring to the surface of the body where the control is introduced, Dirichlet and computed Neumann conditions are implemented for velocity and pressure, respectively.

To simplify notations, define scalar products on the temporal domain [0,*τ*], spatial domain *Ω* and its controlled boundary ***C***

$$\begin{array}{rl} & (\text{a},\text{b})=\int_{\Omega }\text{a}\cdot \text{b}\;d\Omega ,\;\langle \text{a},\text{b}\rangle =\tau^{-1}\int_{0}^{\tau }\int_{\Omega }\text{a}\cdot \text{b}\;d\Omega \;dt,\\ & [\text{d},\text{e}]=\int_{\text{C}}\text{d}\cdot \text{e}\;d\text{C},\;\{\text{d},\text{e}\}=\tau^{-1}\int_{0}^{\tau }\int_{\text{C}}\text{d}\cdot \text{e}\;d\text{C}\;dt, \end{array}$$

where ***a***,***b***∈*Ω*×[0,*τ*] and ***d***,***e***∈***C***×[0,*τ*].

### (b) Surface-velocity control

In this work, three types of surface-velocity control will be studied, i.e. normal control confined to the surface-normal direction, streamwise control constrained to the surface-tangential (streamwise) direction, and a combined control with both surface-normal and streamwise components. To reduce the dimension of the control after temporal–spatial discretization, the temporal and spatial dependence for the three types is decomposed as

2.2 $$\text{u}(\text{C},t)=G(t)u_{n}(\text{C})\text{n},\;\text{u}(\text{C},t)=G(t)u_{m}(\text{C})\text{m}\;\mathrm{and}\;\text{u}(\text{C},t)=G(t){\text{u}}_{\mathrm{nm}}(\text{C}),$$

where *u*_n_(***C***) and *u*_m_(***C***) are the spatial dependence of the control on the normal and streamwise directions, respectively; ***u***_nm_(***C***) is a vector denoting the spatial dependence of the combined control containing a normal component and a streamwise component; ***n*** and ***m*** denote the unit outward normal and streamwise vectors on the controlled surface, respectively; *G*(*t*) is a prescribed temporal-dependence function defined as

2.3 $$G(t)=(1-\exp\left(-\sigma_{1}t^{2}\right))\;\exp(i\omega t),$$

where the first term on the right ensures ***u***(***C***,0)=0 so as to avoid discontinuity in the numerical simulation at *t*=0, considering that a zero initial condition is adopted for the control-induced velocity ***u***; *σ*_1_ is a relaxation factor and *σ*_1_=100 is adopted throughout this work; *ω* acts as the frequency of the control provided that the final time *τ* is large enough.

In the calculation of the control, the temporal dependence is prescribed and the spatial dependence *u*_n_(***C***), *u*_m_(***C***) and ***u***_nm_(***C***) are referred to as the control at frequency *ω*. To evaluate the magnitude of the control, a boundary norm ∥⋅∥_*b*_, also denoted as b-norm in the following, is introduced. This norm is defined as the square root of the square integration of the control along the controlled boundary, e.g. for the normal control

2.4 $$∥u_{n}{∥}_{b}={\left[u_{n},u_{n}\right]}^{1/2}.$$

### (c) Force acting on the aerofoil

Similar to the velocity and pressure terms, the force acting on a solid body can also be decomposed into two components, i.e. an uncontrolled force and a control-induced force, as $\hat{\text{ f}}=\text{ F}+\text{ f}$.

The force acting on a closed surface without boundary control is well known (e.g. [30]):

2.5 $$\text{F}(\text{C})=\int_{\text{C}}(P\text{n}-Re^{-1}\partial_{\text{n}}\text{U})\;d\text{C}.$$

When the control is imposed, the force becomes

2.6 $$\hat{\text{f}}=\int_{\text{C}}(\hat{p}\text{n}-Re^{-1}\partial_{\text{n}}\hat{\text{u}}+\hat{\text{u}}\hat{\text{u}}\;\cdot \;\text{n})\;d\text{C},$$

as derived in appendix A. The three terms integrated on the right-hand side denote the pressure, viscous and thrust forces, respectively. Subtracting the uncontrolled force from the controlled one, one obtains the control-induced force

2.7 $$\text{f}=\int_{\text{C}}(p\text{n}-Re^{-1}\partial_{\text{n}}\text{u})\;d\text{C}.$$

It is noted that the pressure and viscous forces are first-order functions of the control-induced variables, while the thrust force is a second-order function. Therefore, based on the linear assumption (i.e. the magnitude of the control and control-induced variables are small compared with the uncontrolled ones), the thrust force is neglected in the above equation.

Since the uncontrolled force is constant with respect to the control, the following studies will be concentrated on the control-induced force. Because the force is commonly time-dependent, it is more useful to consider the (quasi) mean control-induced force, whose component in direction ***K*** can be written as

2.8 $${\bar{f}}_{\text{K}}=\tau^{-1}\int_{0}^{\tau }\text{f}\;\cdot \;\text{K}R(t)\;dt=\{p\text{n}-Re^{-1}\partial_{\text{n}}\text{u},\text{K}R(t)\},$$

where *R*(*t*)=1−exp^−*σ*_2_(*t*−*τ*)^2^^ is a numerical factor introduced to remove the incompatibility of initial and boundary conditions of the adjoint equation (see §2d) and *σ*_2_=100 is adopted throughout this work. It is seen that, as the final time τ→∞, the influence of *R* on ${\bar{f}}_{\text{ K}}$ tends to be negligible and ${\bar{f}}_{\text{ K}}$ tends to the time-averaged force in direction ***K***. Over the parameters considered in this work, a further increase of *σ* does not result in any significant change of the result. If ***K***=(cos*α*,sin*α*)^*T*^, where *α* denotes the angle of attack, ${\bar{f}}_{\text{ K}}$ is the mean control-induced drag; if ***K***=(−sin*α*, cos*α*)^*T*^, ${\bar{f}}_{\text{ K}}$ becomes the mean control-induced lift.

Since all the variables involved in the governing equations have been non-dimensionalized, the force coefficient is two times the force. To simplify notations, the forces instead of force coefficients are used in the following.

### (d) Sensitivity of force to control

To derive the sensitivity of (mean control-induced) force with respect to control, an adjoint method is applied. Starting from the linearized NS equation and considering integration by parts and the divergence theorem [31], there is

2.9 $$\begin{array}{rl} -\langle {\text{u}}^{*},\partial_{t}\text{u}-L(\text{u})\rangle & =\langle \text{u},\partial_{t}{\text{u}}^{*}+L^{*}({\text{u}}^{*})\rangle -\tau^{-1}({\text{u}}_{\tau },{\text{u}}_{\tau }^{*})+\tau^{-1}({\text{u}}_{0},{\text{u}}_{0}^{*})\\ & \;+\tau^{-1}\int_{0}^{\tau }\int_{\partial \Omega }\text{n}\;\cdot \;[-\text{U}(\text{u}\;\cdot \;{\text{u}}^{*})+\text{u}p^{*}-{\text{u}}^{*}p+Re^{-1}(\nabla \text{u}\;\cdot \;{\text{u}}^{*}-{\nabla \text{u}}^{*}\;\cdot \;\text{u})]\;d\partial \Omega \;dt, \end{array}$$

where ∂*Ω* represents all the boundaries of the computational domain; superscript * denotes the adjoint variables; and u*_τ_ and ***u***_*τ*_ are the adjoint and control-induced velocity vectors at *t*=*τ*, respectively. Here, $L^{*}$ is an adjoint operator of L and correspondingly $\partial_{t}{\text{ u}}^{*}+L^{*}({\text{ u}}^{*})=0$ is the adjoint equation extensively used in investigations of receptivity and non-normality [31–32]. This adjoint operator can be expanded as

$$L^{*}({\text{u}}^{*})={\text{U}\;\cdot \;\nabla \text{u}}^{*}-{\nabla \text{U}\;\cdot \;\text{u}}^{*}-\nabla p^{*}+Re^{-1}\nabla^{2}{\text{u}}^{*}\;\mathrm{and}\;\nabla \;\cdot \;{\text{u}}^{*}=0.$$

On both the inflow and far-field boundaries, zero Dirichlet and computed Neumann conditions are used for adjoint velocity and pressure, respectively; on the outflow, a mixed velocity boundary condition *Re*^−1^∂_***n***_u*+***n*** ⋅ Uu*=0 and a zero Dirichlet pressure condition are implemented [25]; on the controlled boundary, Dirichlet and computed Neumann conditions are used for adjoint velocity and pressure terms, respectively. Considering the sign of the viscous term and the time derivative term, this equation should be initialized at *t*=*τ* and integrated backwards to *t*=0.

Since ***u*** satisfies the linearized NS equation and its initial condition is zero, $\langle {\text{ u}}^{*},\partial_{t}\text{ u}-L(\text{ u})\rangle =0$ and (***u***_0_,u*_0_)=0. Then if the adjoint variables are solutions of the adjoint equation $\partial_{t}{\text{ u}}^{*}+L^{*}({\text{ u}}^{*})=0$ initialized by zero initial condition ${\text{ u}}_{\tau }^{*}=0$, all the terms in (2.9) are zero except the last one. The choice of boundary conditions for the adjoint variables ensures that the integration over the inflow, outflow and far-field boundaries in this last term is zero. Therefore, (2.9) can be reduced to

2.10 $$\{p\text{n}-Re^{-1}\partial_{\text{n}}\text{u},{\text{u}}^{*}\}=\{p^{*}\text{n}-Re^{-1}\partial_{\text{n}}{\text{u}}^{*},\text{u}\}.$$

Combining (2.8) and (2.10), the mean control-induced force can be reformulated as

$${\bar{f}}_{\text{K}}=\{p^{*}\text{n}-Re^{-1}\partial_{\text{n}}{\text{u}}^{*},\text{u}\}$$

by setting the adjoint velocity boundary condition on the controlled boundary as u*=***K****R*(*t*). Here, the relaxation factor *R*(*t*) induces u*(*τ*)=0, which is compatible with the zero initial condition of the adjoint velocity.

Considering the definition of the Gâteaux differential, the gradient of the force with respect to the three types of control can be expressed as

2.11 $$\begin{array}{rl} \nabla_{u_{n}}{\bar{f}}_{\text{K}} & =\tau^{-1}\int_{0}^{\tau }G\text{n}\;\;\cdot \;\;(p^{*}\text{n}-Re^{-1}\partial_{\text{n}}{\text{u}}^{*})\;dt, \end{array}$$

2.12 $$\begin{array}{rl} \nabla_{u_{m}}{\bar{f}}_{\text{K}} & =\tau^{-1}\int_{0}^{\tau }G\text{m}\;\;\cdot \;\;(p^{*}\text{n}-Re^{-1}\partial_{\text{n}}{\text{u}}^{*})\;dt \end{array}$$

2.13 $$\begin{array}{rl} \;\mathrm{and}\;\;\nabla_{{\text{u}}_{\mathrm{nm}}}{\bar{f}}_{\text{K}} & =\tau^{-1}\int_{0}^{\tau }G(p^{*}\text{n}-Re^{-1}\partial_{\text{n}}{\text{u}}^{*})\;dt. \end{array}$$

To calculate these gradients, the uncontrolled flow should be computed first through DNS and then the adjoint variables are solved by integrating the adjoint equation. These gradients are distributed around the surface of the body and can be interpreted as the sensitivity of force to control.

### (e) Control effects

For control with a small enough given b-norm, the mean control-induced force reaches maximum when the distribution of the control coincides with the sensitivity presented above. Therefore, the distribution of the most effective control can be obtained by scaling the sensitivity. Below, the surface-normal control is adopted as an example to demonstrate this relation between the sensitivity and the control.

Any surface control can be decomposed into two components, one parallel with the sensitivity and one normal to it. The parallel component can be calculated by projecting the control to the sensitivity,

$$u_{p}=\frac{\left[u_{n},\nabla_{u_{n}}{\bar{f}}_{\text{K}}\right]}{\left[\nabla_{u_{n}}{\bar{f}}_{\text{K}},\nabla_{u_{n}}{\bar{f}}_{\text{K}}\right]}\nabla_{u_{n}}{\bar{f}}_{\text{K}}.$$

Since $[u_{n},\nabla_{u_{n}}{\bar{f}}_{\text{ K}}]\leq ∥\nabla_{u_{n}}{\bar{f}}_{\text{ K}}{∥}_{b}∥u_{n}{∥}_{b}$, it can be derived that ∥*u*_p_∥_b_≤∥*u*_n_∥_b_. Then according to the definition of the gradient $\nabla_{u_{n}}{\bar{f}}_{\text{ K}}$, the mean control-induced force is

2.14 $${\bar{f}}_{\text{K}}=[u_{n},\nabla_{u_{n}}{\bar{f}}_{\text{K}}]=[u_{p},\nabla_{u_{n}}{\bar{f}}_{\text{K}}].$$

Then if $[u_{n},\nabla_{u_{n}}{\bar{f}}_{\text{ K}}]=0$, *u*_n_ is normal to the sensitivity and the mean control-induced force is zero. If $[u_{n},\nabla_{u_{n}}{\bar{f}}_{\text{ K}}]>0$, *u*_p_ is in the same direction as the sensitivity and ${\bar{f}}_{\text{ K}}=∥\nabla_{u_{n}}{\bar{f}}_{\text{ K}}{∥}_{b}∥u_{p}{∥}_{b}$. If $[u_{n},\nabla_{u_{n}}{\bar{f}}_{\text{ K}}]<0$, *u*_p_ is in the opposite direction to the sensitivity and ${\bar{f}}_{\text{ K}}=-∥\nabla_{u_{n}}{\bar{f}}_{\text{ K}}{∥}_{b}∥u_{p}{∥}_{b}$.

Since ∥*u*_p_∥_b_≤∥*u*_n_∥_b_, the force reaches maximum/minimum when *u*_n_=*u*_p_ (maximum $∥\nabla_{u_{n}}{\bar{f}}_{\text{ K}}{∥}_{b}∥u_{n}{∥}_{b}$ when *u*_n_ is in the same direction as the sensitivity and minimum $-∥\nabla_{u_{n}}{\bar{f}}_{\text{ K}}{∥}_{b}∥u_{n}{∥}_{b}$ when in the opposite direction). Therefore, the b-norm of the sensitivity quantifies the controllability of the force and the distribution of the sensitivity around the body is parallel with the (linearly) most effective control. The corresponding control effect, evaluated by the mean control-induced force, is the product of the b-norm of the sensitivity and the b-norm of the control.

Owing to the linear assumption, this control can be obtained by integrating the adjoint equation once without iteratively calling the governing equations as in nonlinear optimal flow control. The drawback of this approach is that it only yields the most effective control at small b-norms.

## 3. Discretization and convergence

Since the proposed sensitivity/control methodology requires the controlled surface to be smooth, a sharp trailing edge corresponding to infinite curvature does not fit the current methodology (unless eliminating control around the trailing edge by excluding the trailing edge segment from the controlled surface). Thus a relatively thick aerofoil, i.e. NACA0024, is adopted, since its trailing edge can be closed with a smaller curvature and therefore is smoother than thinner aerofoils. Owing to this trailing edge modification, the chord length is reduced to 0.95. The leading edge of the aerofoil is located at (*x*,*y*)=(0,0), the inflow, outflow and far-field boundaries are located at *x*=−30, *x*=145 and *y*=±50, respectively. The whole surface of the aerofoil is considered as the controlled boundary ***C***. Since the aerofoil is symmetric, only positive angles of attack, i.e. 0°≤*α*≤20°, are considered. Therefore, the upper surface of the aerofoil is the suction side and the lower surface is the pressure side. The Reynolds number is defined using the free-stream velocity and the original chord length before rounding the trailing edge. Considering the requirement of computational resources, four relatively small Reynolds numbers, i.e. 500, 1000, 2000 and 5000, where the flow patterns are typical at *α*=10°, are adopted. These Reynolds numbers are much smaller than those for flow around even small-scale wind turbine blades or aircraft wings. However, it is expected that the result of the low Reynolds number flow and the trend at increasing Reynolds number could shed light on the understanding of large Reynolds number flow.

**Figure 1.**
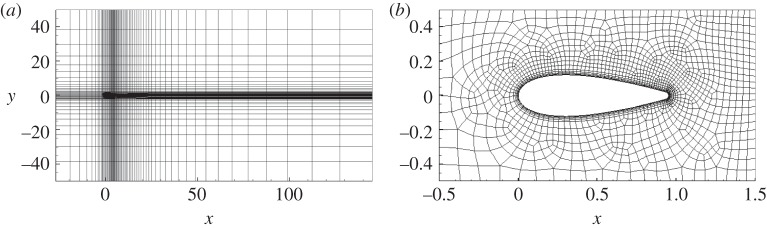
Spectral element decomposition of the computational domain around the modified NACA0024 aerofoil. (*a*) The whole domain and (*b*) the subdomain close to the aerofoil.

Spectral elements employing piecewise continuous nodal-based polynomial expansions are adopted for spatial discretization. Time integration is carried out using a velocity-correction scheme [34]. The same numerics are adopted to integrate the NS equation and the adjoint equation using a well-validated numerical code which has been used in DNS and hydrodynamic stability studies of vortex flow and flow around solid bodies [25,35]. The overall two-dimensional spectral element decomposition consisting of 5815 spectral elements and a close-up view around the aerofoil are shown in figure 1. For three-dimensional simulations, Fourier decomposition is conducted in the spanwise direction, corresponding to implementing periodic spanwise boundary conditions [36].

The initial and boundary conditions for the linearized NS equation and the adjoint equation have been stated in §2. In DNS, the free-stream velocity is specified on the inflow and far-field boundaries; a Dirichlet velocity condition is adopted on the aerofoil; zero Neumann velocity and zero Dirichlet pressure conditions are used on the outflow boundaries. Again a computed Neumann pressure condition is adopted if the velocity condition is of the Dirichlet-type.

As discussed above, the uncontrolled velocity is required in the integration of the linearized NS equation and the adjoint equation. Since the uncontrolled flow is time-dependent, its velocity vector is saved over each prescribed time interval d*T*. In the integration of the linearized NS equation and the adjoint equation, the saved uncontrolled velocity vector is read into memory and reconstructed at every time step through a third-order Lagrangian interpolation [37].

Convergences of the b-norm of the sensitivity of the drag to the normal control are tested at *Re*=5000, the largest Reynolds number considered in this work, as shown in table 1. It is noted that the b-norm of the sensitivity has converged to three significant figures at the polynomial order used to expand each spectral element P=5. Then the polynomial order is fixed at P=5 to check the convergences of the norm with respect to the time interval to save the uncontrolled flow, and the time step to integrate the governing equations, denoted as d*T* and d*t*, respectively. The norm has converged to three significant figures when d*T* is halved from 1.25×10^−2^ to 6.25×10^−3^, or when d*t* is halved from 2.5×10^−4^ to 1.25×10^−4^. Therefore, P=5, d*T*=1.25×10^−2^ and d*t*=2.5×10^−4^ are adopted in the following studies.

**Table 1.** Convergence of the b-norm of the sensitivity of the drag to the normal control in flow past the modified NACA0024 aerofoil. The Reynolds number, angle of attack and final time are *Re*=5000, *α*=10° and *τ*=10, respectively. P, d*T* and d*t* are the polynomial order used to expand each spectral element, the time interval to save the uncontrolled flow and the time step to integrate the governing equations, respectively. ∥⋅∥_b_ denotes the b-norm as defined in (2.4); *u*_n_ represents the surface-normal control; ${\bar{f}}_{\text{ K}}$ is the mean control-induced drag with ***K***=(cos*α*,sin*α*)^*T*^.

P	d*T*	d*t*	$∥\nabla_{u_{n}}{\bar{f}}_{\text{ K}}{∥}_{b}$
2	1.25×10^−2^	2.5×10^−4^	3.6840
3	1.25×10^−2^	2.5×10^−4^	3.3628
4	1.25×10^−2^	2.5×10^−4^	3.3081
5	1.25×10^−2^	2.5×10^−4^	3.3025
6	1.25×10^−2^	2.5×10^−4^	3.3011
7	1.25×10^−2^	2.5×10^−4^	3.3002
5	6.25×10^−3^	2.5×10^−4^	3.3027
5	1.25×10^−2^	1.25×10^−4^	3.3018

**Figure 2.**
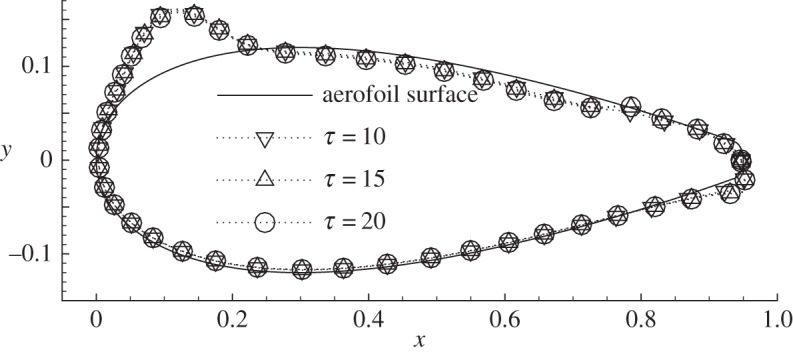
Convergence of the sensitivity of the drag to the normal control at *Re*=5000, *α*=10° and various final times *τ*. The sensitivity has been normalized to have b-norm 0.02 and is plotted based on the aerofoil. The surface-normal deviation of the sensitivity curves from the aerofoil denotes the local magnitude of the sensitivity. A deviation to the aerofoil (in direction ***n***) represents positive sensitivity and vice versa. This convention will be used in all the following plots of distributions of variables along the aerofoil.

As discussed above, a larger final time *τ* is appreciated since it suppresses the numerical relaxation effects associated with *R* and *G*, but apparently it also increases the computational costs. Three final times, i.e. *τ*=10, 15 and 20, are tested and the b-norms of the sensitivities are 3.3025, 3.1517 and 3.18025, respectively. These b-norms do not converge very well owing to the relaxation factors. The convergence of the distribution of the sensitivity with respect to *τ* is further inspected, as shown in figure 2. To illustrate the distribution of sensitivities along the aerofoil, the sensitivities have been normalized to have b-norm 0.02 and therefore the sensitivity curves can be represented as $\text{ C}+0.02\nabla_{u_{n}}{\bar{f}}_{\text{ K}}/{\left[\nabla_{u_{n}}{\bar{f}}_{\text{ K}},\nabla_{u_{n}}{\bar{f}}_{\text{ K}}\right]}^{1/2}$. The surface outward normal ***n*** defines the positive direction of the sensitivity, so sensitivity inside (or outside) the aerofoil is positive (or negative). The distribution of the sensitivity has reached good convergence at *τ*=10 for increasing *τ*, even though its b-norm, which is a function of the relaxation factors and also the final time, is still oscillating with respect to *τ*. Considering that a larger *τ* helps to exclude the transient effects at the beginning of the control as well as the relaxation factor effects, *τ*=20, which is close to the limit of the available computational resources, is adopted in the following investigations if not otherwise stated.

## 4. Results

In this section, the uncontrolled flow is presented in §4a, the magnitude and distribution of the sensitivity are demonstrated in §4b,c, respectively, the control effect of the scaled sensitivity is discussed in §4d and the dominant control mechanisms are identified in §4e, and exploited to control three-dimensional flow in §4f.

### (a) Two-dimensional uncontrolled flow

**Figure 3.**
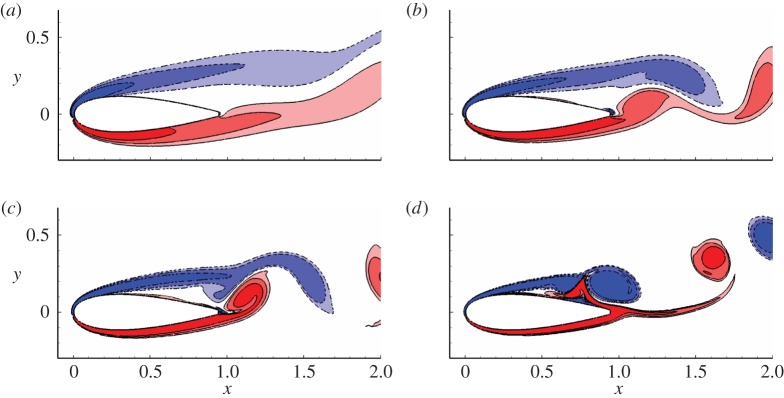
Contours of spanwise vorticity for the uncontrolled flow at *α*=10° and (*a*) *Re*=500, (*b*) *Re*=1000, (*c*) *Re*=2000 and (d**) *Re*=5000, respectively. Contour levels are [−12,−6,−3,3,6,12], as will be used in all the following contour plots if not otherwise stated. (Online version in colour.)

Owing to the computational cost, only the two-dimensional adjoint equation is considered in this work. Therefore, two-dimensional uncontrolled base flows are required to calculate the sensitivity of forces to boundary control. These uncontrolled flows can be obtained by saving the flow field over every time interval d*T* after the solution becomes periodic in two-dimensional DNS (the range of Reynolds numbers and angles of attack considered in this work ensures that a periodic state exists). Figure 3 illustrates the contour of spanwise vorticity for the two-dimensional uncontrolled flow at an angle of attack *α*=10°, time *t*=0 (the time to start saving the uncontrolled flow) and Reynolds numbers *Re*=500, 1000, 2000 and 5000. For Reynolds number higher than 5000, a periodic two-dimensional uncontrolled solution is not obtained at *α*=10°, owing to the high sensitivity of the flow to numerical noise. These four Reynolds numbers are chosen since they correspond to four typical (vortex shedding) flow patterns; at *Re*=500, the unsteady vortex shedding takes place in the wake and the flow close to the aerofoil is almost steady; at *Re*=1000, the unsteady shedding reaches the base of the aerofoil; at *Re*=2000, vortex shedding occurs above the trailing edge of the aerofoil; at *Re*=5000, the shear layer above the suction side breaks into vortices around the middle of the chord.

### (b) Magnitude of the sensitivity

For two-dimensional uncontrolled flow, only two-dimensional sensitivity with spanwise wavenumber zero deserves consideration. This is because, based on the linear assumption, Fourier modes with different spanwise wavenumbers are decoupled and modes with non-zero spanwise wavenumbers induce zero force after integrating along the spanwise direction. This work is concentrated at *Re*=1000 and *α*=10°, where the base flow is two dimensional (asymptotically stable to perturbations with non-zero spanwise wavenumbers), while other Reynolds numbers and angles of attack are also investigated to illustrate the parameter effects.

**Figure 4.**
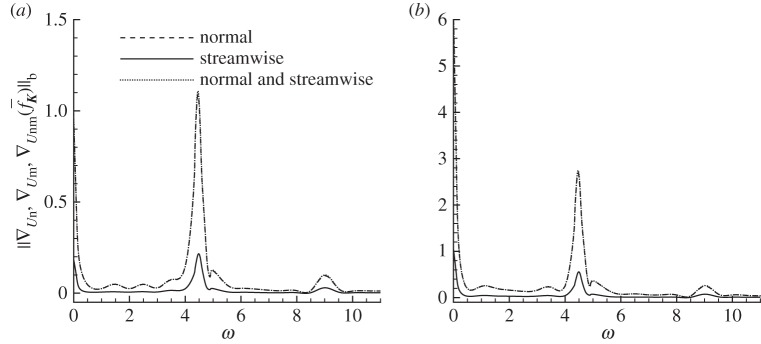
The b-norm of sensitivity of (*a*) drag and (*b*) lift forces with respect to normal, streamwise and combined (normal and streamwise) controls at *Re*=1000 and *α*=10°.

As stated above, three types of control are tested: one restricted to the surface-normal direction (the streamwise component is zero), one constraint to the streamwise direction (normal component is zero), and one with both surface-normal and streamwise components. The sensitivities of forces with respect to these three types of control can be calculated from (2.11)–(2.13). The b-norm of the sensitivity at *Re*=1000 and *α*=10° is presented in figure 4. For all three cases, the b-norm of the sensitivity of lift is much larger than that of drag, indicating that lift can be more effectively modified by boundary control. The b-norm reaches local maxima at frequency *ω*=0, *ω*=4.45 and *ω*=8.90. The first one corresponds to a steady control, the second one is the dominant vortex shedding frequency in the uncontrolled flow and the third one is a higher harmonic. The sensitivity with respect to normal control is much larger than that to the streamwise control and only slightly smaller than the combined control. This observation indicates that, when the control magnitude is small, surface-normal control is much more effective than streamwise control.

In physical experiments, the normal and streamwise controls are generated by different actuators: normal control by jet or synthetic jet and streamwise control by rotation of a segment of the surface, which is intuitively more difficult to implement than that by jet/synthetic jet. Considering the control effectiveness and physical implementations, the following studies will be concentrated on the normal control.

**Figure 5.**
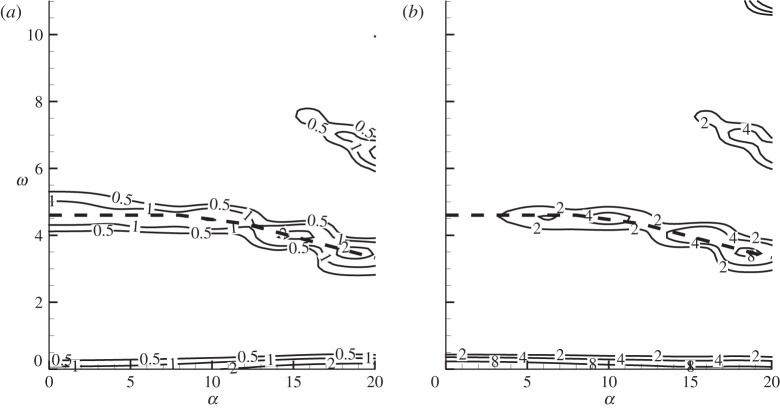
Contours of the b-norm of the sensitivity of (*a*) drag and (*b*) lift forces with respect to normal control at *Re*=1000. The thick dashed lines denote the dominant frequency of the uncontrolled forces.

Firstly, the Reynolds number is fixed at *Re*=1000 and effects of the angle of attack and control frequencies on the sensitivity are investigated, as shown in figure 5. Again the b-norm of the sensitivity of lift is much higher than that of drag. Over the parameters considered, the b-norm reaches a global maximum at *ω*=0 and local maxima at the dominant frequency of the uncontrolled forces (indicated by thick dashed lines in the figure) and higher harmonics. The b-norm increases with the angle of attack, suggesting that the boundary layer separation and shear layer break-up taking place at higher angles of attack result in a more sensitive flow, where the forces can be more effectively modified by boundary control.

**Figure 6.**
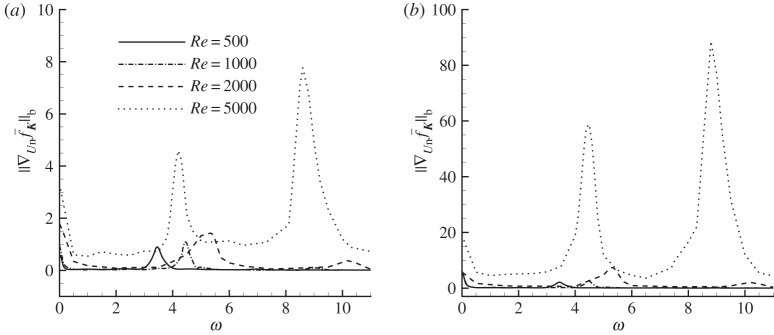
The b-norm of sensitivities of (*a*) drag and (*b*) lift forces with respect to normal control at *α*=10° and various Reynolds numbers.

Then the angle of attack is fixed at *α*=10° to investigate the Reynolds number effect. The b-norm of the sensitivity at four Reynolds numbers, i.e. 500, 1000, 2000 and 5000, where the uncontrolled flow structures are presented in figure 3, are displayed in figure 6. The dominant frequencies for uncontrolled forces are 3.4, 4.5, 4.9 and 4.2 for these four cases. It is seen that the local maxima of the sensitivity take place at zero frequency, dominant frequency of the force and higher harmonics. At higher Reynolds numbers, where the flow becomes more unsteady, featuring flow separation and vortex shedding (figure 3), the forces become increasingly sensitive to boundary control.

### (c) Distribution of the sensitivity

**Figure 7.**
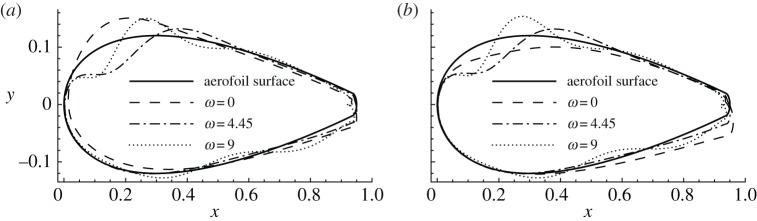
Distribution of sensitivities of (*a*) drag and (*b*) lift forces with respect to normal control at *Re*=1000 and *α*=10°. The sensitivities are normalized to have b-norm 0.02 and are plotted based on the aerofoil.

After discussing the magnitudes of sensitivities, their distributions around the aerofoil are presented. For clarity, the aerofoil surface is divided into four segments: upper leading edge, upper trailing edge, lower leading edge and lower trailing edge, referring to the segment on the suction side from the leading edge to the separation point, the upper surface downstream of the separation point, the forepart of the pressure side and the rear part of the pressure side, respectively. The distributions of three sensitivities whose b-norms reach local maxima with respect to the frequency at *Re*=1000 and *α*=10° are illustrated in figure 7. Similarly to figure 2, the sensitivities are normalized to have b-norm 0.02 and so the curves representing the sensitivities in figure 7 can be expressed as $\text{ C}+0.02\nabla_{u_{n}}{\bar{f}}_{\text{ K}}/{\left[\nabla_{u_{n}}{\bar{f}}_{\text{ K}},\nabla_{u_{n}}{\bar{f}}_{\text{ K}}\right]}^{1/2}$. The deviation of these curves away from the aerofoil denotes the local sign and strength of the sensitivity. For example, the dashed curve in figure 7*a*, which represents the distribution of the sensitivity at *ω*=0, shows strong negative (corresponding to blowing) sensitivities around the upper leading edge, weak positive (corresponding to suction) sensitivities around the upper trailing edge and the lower leading edge, and weak negative sensitivities around the lower trailing edge.

Comparing the distribution of sensitivities of drag and lift at *Re*=1000 and *α*=10°, the steady sensitivities are almost opposite while the two non-zero-frequency sensitivities are positively correlated. This observation suggests that the steady control to increase lift (in the same direction as the sensitivity) would reduce drag, and the control to reduce drag (in the opposite direction to the sensitivity) would increase lift. On the contrary, for the unsteady control, an increase of lift is associated with an increase of drag, and a drag reduction is accompanied by a lift reduction. The most effective control is concentrated on the upper leading edge, where blowing increases lift and reduces drag, and the lower trailing edge, where blowing enhances both lift and drag by increasing the effective angle of attack, similar to the control effect of a flap. The mechanisms of these controls will be analysed in detail in §4e.

**Figure 8.**
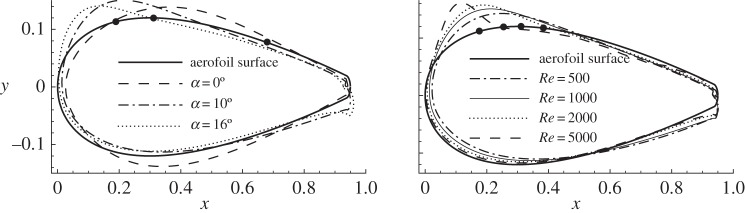
Distribution of sensitivities of drag with respect to normal control, normalized to have b-norm 0.02. (*a*) *ω*=0, *Re*=1000 and various *α*; solid circles denote the separation points at *α*=0°, 10° and 16° from right to left. (*b*) *ω*=0, *α*=10° and various *Re*; solid circles denote the separation points at *Re*=500, 1000, 2000 and 5000 from right to left.

Then the frequency is fixed at *ω*=0, which induces the highest sensitivity, as shown in figure 5, and the variation of sensitivities with the angle of attack is studied, as illustrated in figure 8*a*. The separation points on the suction side of the aerofoil corresponding to a zero shear stress are marked by filled circles on the surface of the aerofoil. The sensitivity of drag is symmetric with respect to the *x*-axis at *α*=0. This sensitivity consists of two parts, suction on the leading edge and blowing upstream of the separation points, both of which tend to ‘squash the surface’ and generate a bluffer body. As the angle of attack increases, the sensitivity becomes asymmetric and more localized around the upper leading edge and the lower trailing edge in the form of blowing. The most sensitive region on the aerofoil is always upstream of the separation point over the parameters considered, as has been observed in several investigations of control of flow separation [14,26,38,39].

The distributions of sensitivity at *ω*=0, *α*=10° and various Reynolds numbers are presented in figure 8*b*. At larger Reynolds numbers, the sensitivity of drag becomes more localized around the upper leading edge upstream of the separation point, similar to the trend at increasing angles of attack. It can be expected that, at even larger Reynolds numbers, the sensitivity will be restricted to an even smaller segment on the aerofoil and therefore facilitates the implementation of control in physical experiments. On the lower surface, the sensitivity does not change significantly at increasing Reynolds numbers. This is unlike the trend at increasing angles of attack, where the forces become more sensitive to trailing edge control.

Practically, the aim of control can be the reduction of drag or the increase of lift. The correlation between the control of lift and control of drag can be calculated as

4.1 $$\rho =\frac{\left[{\text{s}}_{L},{\text{s}}_{D}\right]}{\sqrt{\left[{\text{s}}_{D},{\text{s}}_{D}][{\text{s}}_{L},{\text{s}}_{L}\right]}},$$

where ***s***_L_ and ***s***_D_ denotes the sensitivities of lift and drag, respectively. A positive correlation indicates that the lift and drag are increased (or decreased) simultaneously; a negative correlation suggests that it is possible to increase lift and reduce drag; a zero correlation can be interpreted as the decoupling of the control of lift and drag, e.g. drag is unchanged when lift is increased.

**Figure 9.**
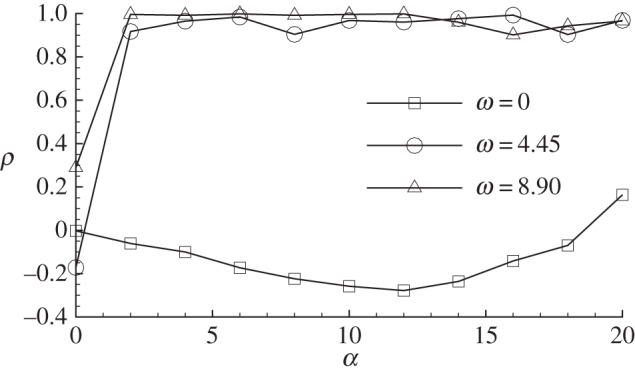
Correlation between sensitivities of lift and drag at *Re*=1000.

From figure 9, the sensitivities of lift and drag are highly correlated at non-zero frequencies. However, for the zero-frequency case, at a small angle of attack, the correlation is zero, because the sensitivity of lift is antisymmetric while the sensitivity of drag is symmetric. At angles of attack around 10°, the correlation is negative, since the upper leading edge becomes the dominantly sensitive region and a suction in this region increases lift and reduces drag. For higher angles of attack, the correlation becomes positive, because the lower trailing edge becomes dominant and a control in this region changes the effective angle of attack and increases (or reduces) lift and drag simultaneously.

### (d) Nonlinear control effects

As discussed in §2e, the control can be obtained by scaling the sensitivity:

4.2 $$u_{n}=b\frac{\nabla_{u_{n}}{\bar{f}}_{\text{K}}}{\sqrt{\left[\nabla_{u_{n}}{\bar{f}}_{\text{K}},\nabla_{u_{n}}{\bar{f}}_{\text{K}}\right]}},$$

where *b* is a scale factor and |*b*| represents the b-norm of the control *u*_n_. If |*b*| is small enough, the controlled force is a linear function of *b*. If *b* exceeds the ‘linear range’, the control-induced flow would not be small enough and the controlled force is no longer linear with respect to *b*. This ‘linear range’ of *b* can be obtained by increasing |*b*| from a small enough value and testing the controlled force through DNS. A momentum coefficient, defined as the square integration of the control around the controlled boundary and the controlled time interval divided by the dynamic pressure of the uncontrolled flow, has been widely used in the literature to evaluate the magnitude of the control. This momentum coefficient is approximately 2*b*^2^ for *ω*=0 and *b*^2^ for *ω*≠0.

The Reynolds number and angle of attack are fixed at *Re*=1000, *α*=10° and two typical control frequencies, *ω*=0 and *ω*=4.45 (the dominant frequency of forces), are adopted. As shown in figure 6, these two frequencies correspond to two local maxima of the sensitivity and so represent the most effective controls.

**Figure 10.**
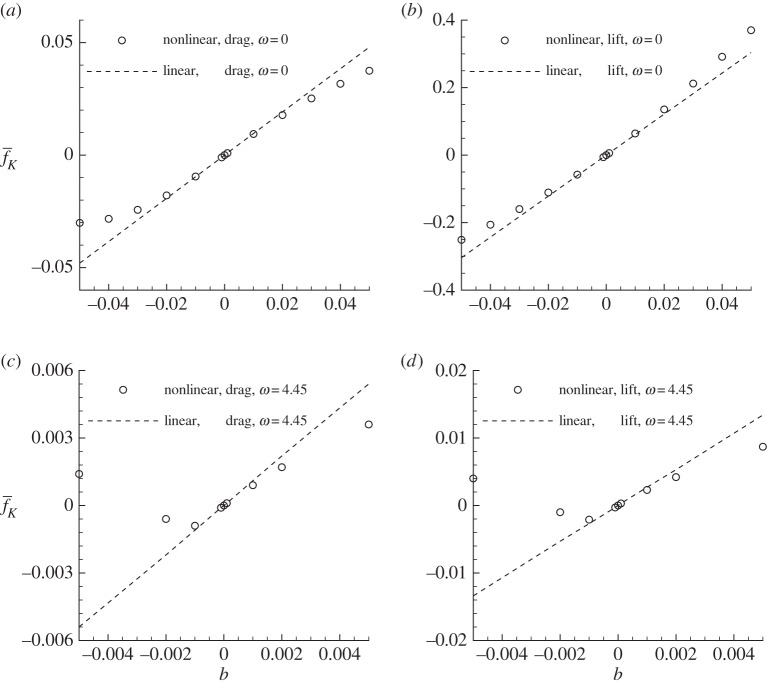
(*a*–*d*) Mean control-induced forces from linear and nonlinear calculations at *ω*=0, *α*=10° and *Re*=1000. *b* is a scale factor, its absolute value is the b-norm of the control and *b*=0 corresponds to the uncontrolled condition.

The nonlinear saturation of the control is shown in figure 10, where the forces induced by the control from ‘linear’ and ‘nonlinear’ calculations are plotted together. The ‘linear’ result can be obtained from (2.14) and (4.2) as

4.3 $${\bar{f}}_{\text{K}}=[u_{n},\nabla_{u_{n}}{\bar{f}}_{\text{K}}]=b∥\nabla_{u_{n}}{\bar{f}}_{\text{K}}{∥}_{b},$$

while the nonlinear result is obtained by subtracting the mean uncontrolled force, calculated through DNS with no-slip boundary conditions on the aerofoil, from the mean controlled force, calculated through DNS with boundary condition *G*(*t*)*u*_n_(***C***)***n*** on the aerofoil.

From the steady control as shown in figure 10*a*,*b*, for small |*b*|, the linear and nonlinear results agree very well for all the cases considered. As |*b*| increases, the nonlinear results deviate from the linear prediction and the linear range of the control with respect to lift is much higher than that to drag. The nonlinear saturation reduces the linear predicted control effects, except that, at positive *b* for the control of lift, the nonlinear interaction of the control-induced flow strengthens the control effect. Considering that the mean uncontrolled drag and lift forces are 0.108 and 0.082, respectively, at *b*=0.02, the control changes drag by 20%. It is noted that at *b*=0.02, corresponding to a momentum coefficient of 0.0008, the maximum surface-normal control velocity is less than 0.036, suggesting that a well-distributed control force is effective to modify the forces even in the linear range.

The unsteady control at *ω*=4.45 is presented in figure 10*c*,*d*. At around *b*=0.002, the nonlinear control effects start to deviate from the linear results for both the lift and drag forces; at *b*=−0.005, the control effects are reversed from the linear expectation. This is because the two controls at *b*=0.005 and *b*=−0.005 have the same distribution (with opposite phase), and the controlled flows are ‘locked-in’ to the control and exhibit the same flow patterns but with opposite phases. Therefore, even though the two controls, one at *ω*=0 and another at *ω*=4.45, have close control effectiveness as indicated from the b-norm of the sensitivity, the steady control has a much wider ‘linear range’ and acts as an effective control even at large magnitudes. On the contrary, the unsteady control is only effective when the control magnitude is one order smaller owing to the nonlinear lock-in effect. Thus the following study will be concentrated on the steady control.

**Figure 11.**
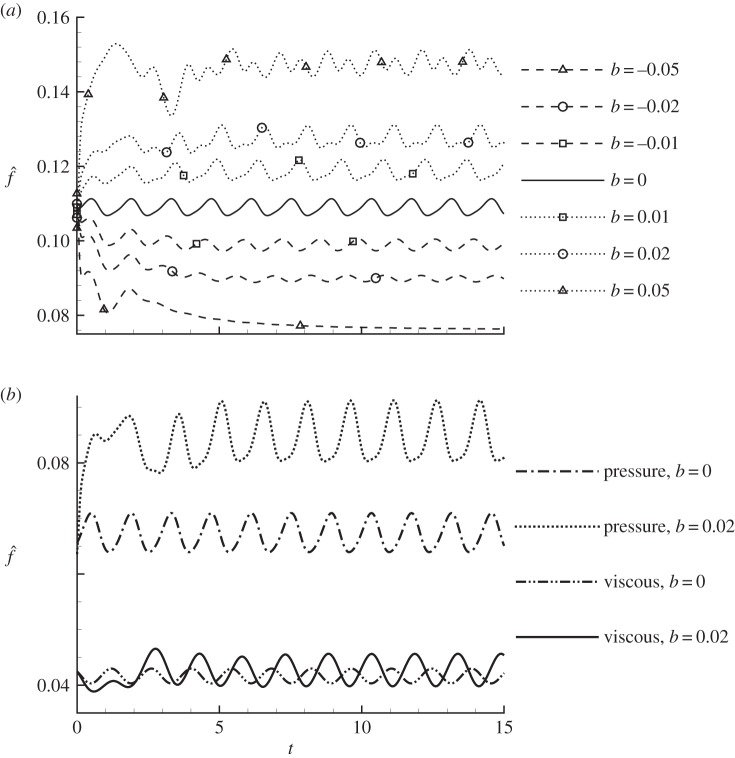
Nonlinear development of the controlled (*a*) total drag and (*b*) decomposed drag components at *ω*=0, *α*=10° and *Re*=1000. The control is scaled from the sensitivity with respect to drag.

The control-induced force shown in figure 10 only represents the mean force, while the time development of the controlled force is shown in figure 11*a*. Since the control effects on lift and drag are similar, only the controlled drag is presented in the rest of this section. After an initial transient period, for all the cases considered, the controlled forces reach periodic states. Clearly, *b*=0 corresponds to the uncontrolled case. At *b*=−0.05, the controlled drag is almost steady, and at increasing *b*, the drag increases in both mean value and oscillation magnitude, suggesting that the mean drag and flow unsteadiness are controlled simultaneously.

The controlled forces are decomposed into pressure, viscous and thrust forces (equation (2.6)), as shown in figure 11*b*. Owing to the choice of small values of |*b*|, the thrust force, which is a second-order function of the control, is negligible (less than 1% of the total force over the cases studied) and is not plotted. It is seen that the change of the viscous force is much smaller than that of the pressure force, revealing that the control modifies the force mainly by changing the pressure distribution around the aerofoil.

**Figure 12.**
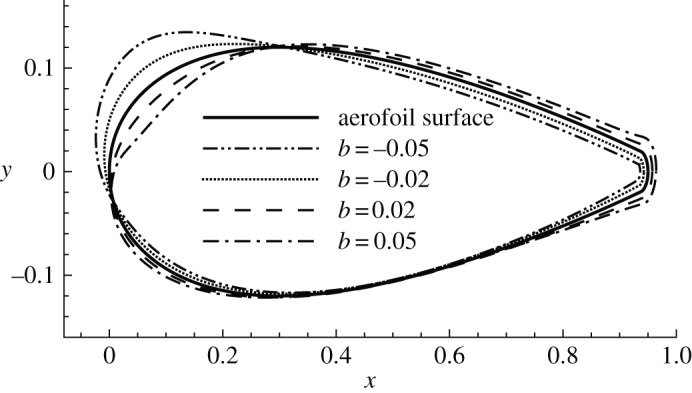
Distributions of control-induced pressure based on the aerofoil at *ω*=0, *α*=10° and *Re*=1000, averaged over 10≤*t*≤20. The control is scaled from the sensitivity with respect to drag.

Figure 12 illustrates the distribution of the control-induced pressure. Similar to the sensitivity, the controlled pressure curves can be expressed as ***C***+0.1*p****n***, where the factor 0.1 is used to scale the control-induced pressure so that the pressure curves can be plotted based on the aerofoil. Here, the pressure distribution is time-averaged over 10≤*t*≤20 instead of the full time period in order to exclude the transient effects at the beginning of the control (figure 11). Therefore, if the pressure curve is inside the aerofoil (aligning with ***n***), the control-induced pressure is positive or the control increases the pressure, and vice versa. For example, at *b*=0.05, the pressure curve around the upper leading edge is inside the aerofoil, which can be interpreted as an increase of pressure due to control. At negative values of *b*, which reduce drag, the pressure around the upper leading edge reduces and pressure around the trailing edge increases. Details of the control mechanism and pressure distributions will be discussed in §4e.

**Figure 13.**
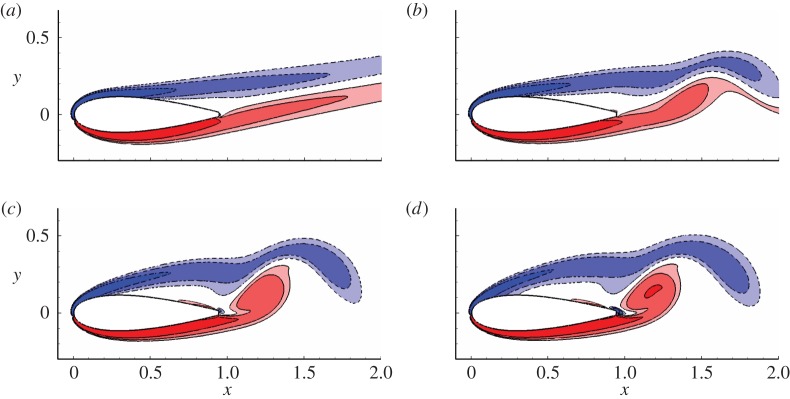
Contours of vorticity at *t*=20, *Re*=1000, *α*=10°, *ω*=0 and scale factor (*a*) *b*=−0.05, (*b*) *b*=−0.02, (*c*) *b*=0.02 and (*d*) *b*=0.05. The control is scaled from the sensitivity with respect to drag. (Online version in colour.)

The flow patterns under the control with respect to drag are illustrated in figure 13. As observed in the development of controlled forces in figure 11, the flow tends to be steady at *b*=−0.05. At negative *b*, the shear layer above the upper surface is attracted to the boundary by the suction control and the separation is delayed. At positive *b*, the shear layer is blown away from the boundary (attracted to bend towards the surface by control around the upper trailing edge) and the separation is promoted. Owing to the control effects on separation, vortex shedding is correspondingly suppressed (or promoted) at negative (or positive) *b*, further indicating that the mean drag and flow fluctuations are controlled simultaneously.

### (e) Control mechanisms

**Figure 14.**
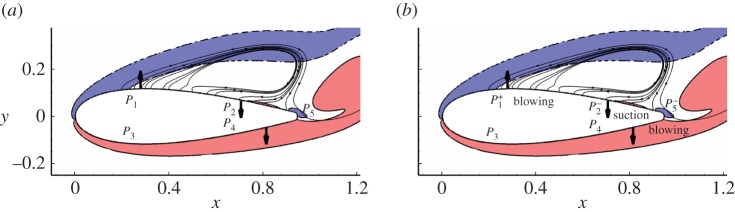
Schematic plots of (*a*) uncontrolled and (*b*) controlled flow. The control is scaled from the sensitivity to drag at *Re*=1000, *α*=10°, *ω*=0 and *b*=0.05. The arrows denote the direction of the control. *P*_1_, *P*_2_, *P*_3_, *P*_4_ and *P*_5_ represent the uncontrolled pressure at the upper leading edge, upper trailing edge, lower leading edge, lower trailing edge and base regions, respectively. Superscripts + and − represent an increase and decrease in the pressure owing to the boundary control, respectively. Solid and dashed lines are contour lines of spanwise vorticity −6 and 6, respectively. Thin lines with arrows are streamlines around the suction side. (Online version in colour.)

To analyse the control mechanisms, the control with respect to drag at *Re*=1000, *α*=10°, *ω*=0 and *b*=0.05 is adopted. The pressure change, control velocity, flow vorticity contours and streamlines are plotted together in figure 14. The aerofoil is divided into five parts, upper leading edge, upper trailing edge, lower leading edge, lower trailing edge and base regions. The first four parts have been defined before, and the last one referring to the trailing edge region is introduced since the pressure in this region is critical to drag. The uncontrolled pressures around these five regions are represented by *P*_1_, *P*_2_, *P*_3_, *P*_4_ and *P*_5_, respectively, as marked in figure 14*a*. An increase or decrease in these pressures owing to the control is denoted by superscript + and −, respectively.

It is seen that the control mainly consists of three parts, i.e. suction around the upper trailing edge, blowing around the lower trailing edge and blowing around the upper leading edge. The first component attracts the separating shear layer associated with low pressure to the surface and therefore reduces pressure on the upper surface downstream of the separation point. The second one pushes the lower shear layer away, promotes separation and increases the effective angle of attack. The third one is located in the most sensitive region for both lift and drag as identified above, i.e. the segment on the upper surface upstream of the separation point, where the pressure reaches local minimum. The control imposed slightly upstream of the separation point has been shown to be effective to eliminate separation and unsteadiness in both DNS and experimental works for boundary layer, aerofoil and cylinder flows [10,14,38]. In this low-pressure region, blowing increases the local pressure and suction reduces the pressure.

### (f) Three-dimensional control effects of upper leading edge blowing/suction

The study presented above has been focused on the case with *Re*=1000 and *α*=10°, where the uncontrolled flow is obtained from two-dimensional DNS. In this section, the angle of attack is fixed at *α*=10° and higher Reynolds numbers are adopted to activate three-dimensional development of the flow. In the integration of the three-dimensional adjoint equation, the adjoint variables and subsequently the sensitivity diverge owing to the chaotic dynamics of the uncontrolled base flow, as observed in the present study as well as in a flow past a cylinder at *Re*=500 [30]. Therefore, in three-dimensional conditions, the control mechanisms identified in two-dimensional studies are exploited to obtain the control without solving the adjoint equation. As discussed above, suction around the upper leading edge upstream of the separation point can be expected to reduce drag and enhance lift effectively. Therefore, suction/blowing located around the upper leading edge upstream of the separation point is adopted as the control. As captured in figure 8*b*, this control maximizes at (*x*,*y*)=(0.21,0.12) and spans from (*x*,*y*)=(0.02,0.04) to (*x*,*y*)=(0.42,0.11) at *Re*=2000, and maximizes at (*x*,*y*)=(0.16,0.11) and spans from (*x*,*y*)=(0.01,0.03) to (0.24,0.12) at *Re*=5000.

The controlled and uncontrolled flows can be obtained through three-dimensional DNS, where the spanwise boundary conditions are set to periodic, and conditions on all the other boundaries are the same as in two-dimensional simulations. The spanwise length is set to *L*=1, which has been tested to be long enough to accommodate three-dimensional instabilities, and 64 Fourier modes with spanwise wavenumbers 0,2*π*,4*π*,6*π*,…,126*π*, are calculated.

**Figure 15.**
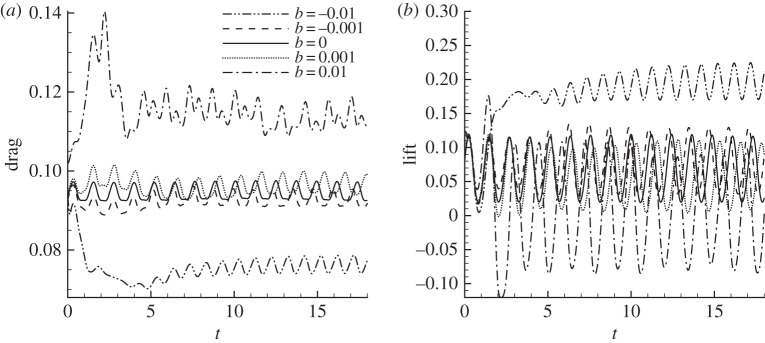
Three-dimensional control effects on (*a*) drag and (*b*) lift at *Re*=2000 and *α*=10°. The control is imposed around the upper leading edge in the form of blowing (positive *b*) or suction (negative *b*).

The controlled drag/lift forces at *Re*=2000 are illustrated in figure 15. At *b*=−0.01, where the maximum control in the form of suction is 5% of the free-stream velocity, the drag is reduced by 20% and the lift is enhanced by 200%. As for the reduction (or enhancement) of drag (or lift), the oscillation of the force reduces correspondingly. This simultaneous control of the force magnitude and oscillation has also been observed in two-dimensional conditions. It is worth noting that there is a strong transient drag increase at positive values of *b*, suggesting potential applications of this control in vehicle deceleration or braking.

**Figure 16.**
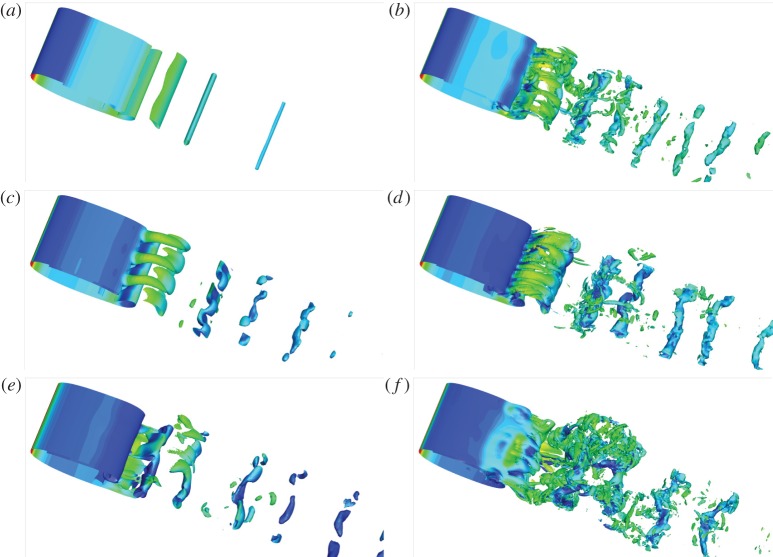
Iso-surfaces of spanwise vorticity −10 and 10 (coloured by pressure) of the controlled flow at *ω*=0, *α*=10°, *t*=10 and (*Re*,*b*)=(2000, −0.01), (5000, −0.01), (2000, 0), (5000, 0), (2000, 0.01) and (5000, 0.01) for (*a*–*f*), respectively. (Online version in colour.)

The corresponding three-dimensional structures at *Re*=2000 and *Re*=5000 are illustrated in figure 16. It is noted that, for the uncontrolled flow, the dominant spanwise wavenumber is 6*π* at *Re*=2000, while at *Re*=5000 the wake flow becomes fully turbulent and no dominant three-dimensional waves can be identified. For *b*<0, the suction around the upper leading edge suppresses boundary-layer separation and reduces unsteadiness (e.g. three-dimensional developments and two-dimensional vortex shedding). At *b*=−0.01 and *Re*=2000, the controlled flow becomes almost steady and stable to three-dimensional perturbations, as can be seen in figure 16*c* and proved in an extra set of Floquet analyses. For *b*>0, the flow becomes increasingly unsteady and turbulent owing to the promotion of separation and vortex shedding.

## 5. Conclusion

The sensitivity of forces with respect to boundary control in an aerofoil flow is investigated. Instead of studying control generated by a finite number of actuators, this work is concentrated on the optimal distributions of the control around the aerofoil. This distributed control has been recently investigated in bluff body flow using streamwise body-surface control [40,41]. In the condition that the control velocity is small enough, the most effective control and the expected (linear) control effects can be obtained by scaling the sensitivity. The calculation of this control involves a single integration of the adjoint of the linearized NS equation, without iterative calling of the governing equations as used in nonlinear optimal control [40,42]. The uncontrolled velocity is required in the integration of the adjoint, and therefore a DNS of the uncontrolled flow is conducted and the velocity history is saved.

A NACA0024 aerofoil is adopted and is closed with a round trailing edge to satisfy the smooth-surface constraint of the sensitivity methodology, even though a sharp trailing edge can be accommodated by suppressing control around the trailing edge. The study is focused on the case with *Re*=1000 and *α*=10°, at which the uncontrolled flow is two-dimensional, and is also extended to other parameters, e.g. 500≤*Re*≤5000 and 0≤*α*≤20°. The three-dimensional effects are investigated by adopting Fourier expansion in the spanwise direction at *Re*≥2000 and *α*=10°, where the uncontrolled flow is unstable to three-dimensional disturbances.

The sensitivities of forces with respect to streamwise, normal and combined (both streamwise and normal) controls are calculated. The magnitude of the sensitivity is measured by a b-norm, defined as the square root of the square integration of the sensitivity around the aerofoil. The sensitivity b-norm quantifies the controllability of the force, and the distribution of the sensitivity represents a control. Over all the parameters considered, the sensitivity of lift is much larger than that of drag. It is also noted that the sensitivity to streamwise control is much smaller than that to normal control and that the sensitivity to the combined control is only slightly larger than that to normal control. Considering that the normal and streamwise controls are generated by completely different physical actuators and that the streamwise control is far less effective than the normal control, this work focuses on the normal control.

The b-norm of the sensitivity reaches local maxima at the zero frequency, the dominant frequency of the uncontrolled forces and higher harmonics. At the first frequency, the sensitivities of drag and lift are negatively correlated while at the other two they are positively correlated. As the Reynolds number or angle of attack increases, the shape of the sensitivity becomes increasingly localized around the upper leading edge and lower trailing edge. This localization indicates that, at large angle of attack or Reynolds number, an effective control can be generated by a limited number of actuators installed around the most sensitive segment on the aerofoil.

A control most effective to modify the force in the linear sense can be obtained by scaling the sensitivity. The linear range of the control, in which the control effect matches the prediction from the linear sensitivity study, is tested through DNS. The linear range for unsteady controls is one order smaller than that for steady controls, suggesting that, for the linear control mechanism, a steady control is much more effective than an unsteady control. For the steady control, at b-norm 0.02, corresponding to a maximum surface-normal velocity of 3.6% of the free-stream velocity, the control reduces drag by up to 20% or increases lift by up to 140% at *Re*=1000. Decomposing the force into pressure, viscous and thrust terms, it is seen that the control effect mainly acts on the pressure term. Therefore, the control mechanism relies largely on the redistribution of pressure around the aerofoil.

The region upstream of the separation point around the upper leading edge is identified as the most sensitive region [10,14,38], and suction in this region reduces drag and increases lift. This mechanism is further exploited to generate the control for three-dimensional turbulent flow, where the sensitivity diverges owing to the chaotic dynamics of the flow. An upper leading edge suction with a momentum coefficient of 0.01 is found to reduce drag by up to 20% and increase lift by 200%. In both two- and three-dimensional controls, drag reduction is associated with the reduction of flow unsteadiness and subsequently the oscillation of forces.

## Appendix A. Derivation of equation (2.6)

The velocity vector around the surface of a solid body can be decomposed in the Cartesian coordinates as ${\hat{u}}_{x}$, ${\hat{u}}_{y}$ and ${\hat{u}}_{z}$, or as a surface-normal component (${\hat{u}}_{n}$), a streamwise component (${\hat{u}}_{m}$) and a spanwise component (${\hat{u}}_{z}$):

A 1 $$\hat{\text{u}}={\hat{u}}_{x}\text{i}+{\hat{u}}_{y}\text{j}+{\hat{u}}_{z}\text{k}={\hat{u}}_{n}\text{n}+{\hat{u}}_{m}\text{m}+{\hat{u}}_{z}\text{k},$$

where ***i***, ***j***, ***k***, ***n*** and ***m*** are units in the *x*, *y*, *z*, outward surface-normal and streamwise directions, respectively. ***n*** and ***m*** can be decomposed as ***n***=*n*_*x*_***i***+*n*_*y*_***j*** and ***m***=*m*_*x*_***i***+*m*_*y*_***j***, where *m*_*x*_=*n*_*y*_ and *m*_*y*_=−*n*_*x*_. Correspondingly, there are ${\hat{u}}_{n}={\hat{u}}_{x}n_{x}+{\hat{u}}_{y}n_{y}$, ${\hat{u}}_{m}={\hat{u}}_{x}m_{x}+{\hat{u}}_{y}m_{y}$, ${\hat{u}}_{x}={\hat{u}}_{m}m_{x}+{\hat{u}}_{n}n_{x}$ and ${\hat{u}}_{y}={\hat{u}}_{m}m_{y}+{\hat{u}}_{n}n_{y}$. In this work, it is assumed that the controlled boundary is a concave and closed surface (curve) and ${\hat{u}}_{x}$, ${\hat{u}}_{y}$, *m*_*x*_ and *m*_*y*_ are differentiable.

The force acting on the surface of the solid body ***C*** can be written as

A 2 $$\hat{\text{f}}=\int_{\text{C}}(\hat{p}\text{n}-\hat{\tau }\;\cdot \;\text{n}+\hat{\text{u}}{\hat{u}}_{n})\;dS,$$

where the three terms integrated represent pressure, viscous and thrust terms; $\hat{\tau }$ is the viscous shear stress and

A 3 $$\hat{\tau }\;\cdot \;\text{n}=Re^{-1}\left[\begin{array}{cc} 2\partial_{x}{\hat{u}}_{x} & \partial_{y}{\hat{u}}_{x}+\partial_{x}{\hat{u}}_{y}\\ \partial_{y}{\hat{u}}_{x}+\partial_{x}{\hat{u}}_{y} & 2\partial_{y}{\hat{u}}_{y} \end{array}\right]\;\cdot \;\left[\begin{array}{c} n_{x}\\ n_{y} \end{array}\right].$$

Through standard algebraic manipulations, there is

A 4 $$n_{x}\partial_{x}{\hat{u}}_{x}+n_{y}\partial_{x}{\hat{u}}_{y}=\partial_{x}{\hat{u}}_{n}-{\hat{u}}_{m}(m_{x}\partial_{x}n_{x}+m_{y}\partial_{x}n_{y}).$$

In this derivation, $n_{x}\partial_{x}n_{x}+n_{y}\partial_{x}n_{y}=\partial_{x}(n_{x}^{2}+n_{y}^{2})/2=0$ has been used. Similarly, there are

A 5 $$n_{x}\partial_{y}{\hat{u}}_{x}+n_{y}\partial_{y}{\hat{u}}_{y}=\partial_{y}{\hat{u}}_{n}-{\hat{u}}_{m}(m_{x}\partial_{y}n_{x}+m_{y}\partial_{y}n_{y}).$$

Substitute (A4) and (A5) into (A3) to reach

A 6 $$\hat{\tau }\;\cdot \;\text{n}=Re^{-1}[\partial_{\text{n}}\hat{\text{u}}+\nabla {\hat{u}}_{n}-{\hat{u}}_{m}\text{m}\;\cdot \;{\left(\nabla \text{n}\right)}^{T}].$$

Then substitute (A6) into (A2) to obtain

A 7 $$\hat{\text{f}}=\int_{\text{C}}(\hat{p}\text{n}-Re^{-1}\partial_{\text{n}}\hat{\text{u}}+\hat{\text{u}}{\hat{u}}_{n})\;dS+Re^{-1}\int_{\text{C}}-\nabla {\hat{u}}_{n}+{\hat{u}}_{m}\text{m}\;\cdot \;{\left(\nabla \text{n}\right)}^{T}\;dS.$$

In the following, it will be presented that the second term after the equality, which involves an integration over the closed curve ***C***, is zero.

From the divergence-free condition, it can be derived that

$$\partial_{\text{n}}{\hat{u}}_{n}+\partial_{\text{m}}{\hat{u}}_{m}+{\hat{u}}_{n}(\partial_{x}n_{x}+\partial_{y}n_{y})+{\hat{u}}_{m}(\partial_{x}m_{x}+\partial_{y}m_{y})=0.$$

Therefore, one has

A 8 $$\begin{array}{rl} \int_{\text{C}}\nabla {\hat{u}}_{n}\;dS & =\int_{\text{C}}\text{n}\partial_{\text{n}}{\hat{u}}_{n}+\text{m}\partial_{\text{m}}{\hat{u}}_{n}\;dS=\int_{\text{C}}\partial_{\text{m}}({\hat{u}}_{n}\text{m}-{\hat{u}}_{m}\text{n})\;dS\\ & \;\int_{\text{C}}-{\hat{u}}_{n}[\partial_{\text{m}}\text{m}+(\partial_{x}n_{x}+\partial_{y}n_{y})\text{n})]+{\hat{u}}_{m}[\partial_{\text{m}}\text{n}-(\partial_{x}m_{x}+\partial_{y}m_{y})\text{n}]\;dS. \end{array}$$

Since the perturbation boundary is assumed to be a closed surface, there is

A 9 $$\int_{\text{C}}\partial_{\text{m}}({\hat{u}}_{n}\text{m}-{\hat{u}}_{m}\text{n})\;dS=0.$$

Through standard algorithm manipulations, there is

A 10 $$\partial_{\text{m}}\text{m}+(\partial_{x}n_{x}+\partial_{y}n_{y})\text{n}=0$$

and

A 11 $$\partial_{\text{m}}\text{n}-(\partial_{x}m_{x}+\partial_{y}m_{y})\text{n}=\text{m}\;\cdot \;{\left(\nabla \text{n}\right)}^{T}.$$

Substitute (A9)–(A11) into (A8) to reach

A 12 $$\int_{\text{C}}-\nabla {\hat{u}}_{n}+{\hat{u}}_{m}\text{m}\;\cdot \;{\left(\nabla \text{n}\right)}^{T}\;dS=0.$$

Substitute (A12) into (A7) to obtain the controlled force

$$\hat{\text{f}}=\int_{\text{C}}(\hat{p}\text{n}-Re^{-1}\partial_{\text{n}}\hat{\text{u}}+\hat{\text{u}}{\hat{u}}_{n})\;dS.$$

## References

[1] Choi H, Jeon W, Kim J 2008 Control of flow over a bluff body. Annu. Rev. Fluid Mech. 40, 113–139. (doi:10.1146/annurev.fluid.39.050905.110149)

[2] Xu J, Dong S, Maxey M, Karniadakis G 2007 Turbulent drag reduction by constant near-wall forcing. J. Fluid Mech. 582, 79–101. (doi:10.1017/S0022112007005460)

[3] Wu C, Wang L, Wu J 2007 Suppression of the von Kármán vortex street behind a circular cylinder by a travelling wave generated by a flexible surface. J. Fluid Mech. 574, 365–391. (doi:10.1017/S0022112006004150)

[4] Artana G, Sosa R, Moreau E, Touchard G 2003 Control of the near-wake flow around a circular cylinder with electrohydrodynamic actuators. Exp. Fluids 35, 580–588. (doi:10.1007/s00348-003-0704-z)

[5] Glezer A, Amitay M 2002 Synthetic jets. Annu. Rev. Fluid Mech. 34, 503–529. (doi:10.1146/annurev.fluid.34.090501.094913)

[6] Arcas D, Redekopp L 2004 Aspects of wake vortex control through base blowing/suction. Phys. Fluids 16, 452–456. (doi:10.1063/1.1637354)

[7] Sevilla A, Martìnez-Bazán C 2004 Vortex shedding in high Reynolds number axisymmetric bluff-body wakes: local linear instability and global bleed control. Phys. Fluids 16, 3460–3469. (doi:10.1063/1.1773071)

[8] Delaunay Y, Kaiktsis L 2001 Control of circular cylinder wakes using base mass transpiration. Phys. Fluids 13, 3285–3302. (doi:10.1063/1.1409968)

[9] Kametani Y, Fukagata K 2011 Direct numerical simulation of spatially developing turbulent boundary layers with uniform blowing or suction. J. Fluid Mech. 681, 154–172. (doi:10.1017/jfm.2011.219)

[10] Kim J, Choi H 2005 Distributed forcing of flow over a circular cylinder. Phys. Fluids 17, 033103 (doi:10.1063/1.1850151)

[11] Darekar RM, Sherwin SJ 2001 Flow past a square-section cylinder with a wavy stagnation face. J. Fluid Mech. 426, 263–295. (doi:10.1017/S0022112000002299)

[12] Park H, Lee D, Jeon W, Hahn S, Kim J, Kim J, Choi J, Choi H 2006 Drag reduction in flow over a two-dimensional bluff body with a blunt trailing edge using a new passive device. J. Fluid Mech. 563, 389–414. (doi:10.1017/S0022112006001364)

[13] Wu J, Lu X, Fan M, Denny A, Wu J 1998 Post-stall flow control on an airfoil by local unsteady forcing. J. Fluid Mech. 371, 21–58. (doi:10.1017/S0022112098002055)

[14] Raju R, Mittal R, Cattafesta L 2008 Dynamics of airfoil separation control using zero-net mass-flux forcing. AIAA J. 46, 3103–3115. (doi:10.2514/1.37147)

[15] Kotapati R, Mittal R, Cattafesta L 2006 Numerical experiments in synthetic jet based separation control. In *Proc. 44th AIAA Aerospace Science Meeting and Exhibit, Reno, NV, 9–12 January 2006.* Reston, VA: Aerospace Research Central.

[16] Hong G 2006 Effectiveness of micro synthetic jet actuator enhanced by flow instability in controlling laminar separation caused by adverse pressure gradient. Sens. Actuators A 132, 607–615. (doi:10.1016/j.sna.2006.02.040)

[17] Glezer A 2011 Some aspects of aerodynamic flow control using synthetic-jet actuation. Phil. Trans. R. Soc. A 369, 1476–1494. (doi:10.1098/rsta.2010.0374) 21382826 10.1098/rsta.2010.0374

[18] Jeon S, Choi J, Jeon W, Choi H, Park J 2004 Active control of flow over a sphere for drag reduction at a subcritical Reynolds number. J. Fluid Mech. 517, 113–129. (doi:10.1017/S0022112004000850)

[19] James D, Truong Q 1972 Wind load on cylinder with spanwise protrusion. J. Eng. Mech. Div. 98, 1573–1589.

[20] Giannetti F, Luchini P 2007 Structural sensitivity of the first instability of the cylinder wake. J. Fluid Mech. 581, 167–197. (doi:10.1017/S0022112007005654)

[21] Marquet O, Sipp D, Jacquin L 2008 Sensitivity analysis and passive control of cylinder flow. J. Fluid Mech. 615, 221–252. (doi:10.1017/S0022112008003662)

[22] Pralits JO, Brandt L, Giannetti F 2010 Instability and sensitivity of the flow around a rotating circular cylinder. J. Fluid Mech. 650, 513–536. (doi:10.1017/S0022112009993764)

[23] Schmid PJ 2007 Nonmodal stability theory. Annu. Rev. Fluid Mech. 39, 129–162. (doi:10.1146/annurev.fluid.38.050304.092139)

[24] Mao X, Blackburn HM, Sherwin SJ 2012 Optimal inflow boundary condition perturbations in steady stenotic flows. J. Fluid Mech. 705, 306–321. (doi:10.1017/jfm.2012.58)

[25] Mao X, Blackburn HM, Sherwin SJ 2013 Calculation of global optimal initial and boundary perturbations for the linearised incompressible Navier–Stokes equations. J. Comput. Phys. 235, 258–273. (doi:10.1016/j.jcp.2012.10.049)

[26] Boujo E, Gallaire F 2014 Manipulating flow separation: sensitivity of stagnation points, separatrix angles and recirculation area to steady actuation. Proc. R. Soc. A 470, 20140365 (doi:10.1098/rspa.2014.0365) 25294968 10.1098/rspa.2014.0365 PMC4156148

[27] Meliga P, Boujo E, Pujals G, Gallaire F 2014 Sensitivity of aerodynamic forces in laminar and turbulent flow past a square cylinder. Phys. Fluids 26, 104101 (doi:10.1063/1.4896941)

[28] Jameson A, Ou K 2010 Optimization methods in computational fluid dynamics. In *Encyclopedia of aerospace engineering* (eds R Blockley, W Shyy). New York, NY: John Wiley & Sons.

[29] Karniadakis GE, Israeli M, Orszag SA 1991 High-order splitting methods for the incompressible Navier–Stokes equations. J. Comput. Phys. 97, 414–443. (doi:10.1016/0021-9991(91)90007-8)

[30] Wang Q, Gao J 2013 The drag-adjoint field of a circular cylinder wake at Reynolds numbers 20, 100 and 500. J. Fluid Mech. 730, 145–161. (doi:10.1017/jfm.2013.323)

[31] Barkley D, Blackburn HM, Sherwin SJ 2008 Direct optimal growth analysis for timesteppers. Int. J. Num. Meth. Fluids 57, 1435–1458. (doi:10.1002/fld.1824)

[32] Hill D 1995 Adjoint systems and their role in the receptivity problem for boundary layers. J. Fluid Mech. 292, 183–204. (doi:10.1017/S0022112095001480)

[33] Giannetti F, Luchini P 2003 Receptivity of the circular cylinder’s first instability. In *Proc. 5th Eur. Fluid Mech. Conf. Toulouse, France, 24–28 August 2003*.

[34] Sherwin SJ 1997 Hierarchical *hp* finite elements in hybrid domains. Finite Elem. Anal. Des. 27, 109–119. (doi:10.1016/S0168-874X(97)00008-5)

[35] Mao X, Blackburn HM, Sherwin SJ 2015 Nonlinear optimal suppression of vortex shedding from a circular cylinder. J. Fluid Mech. 775, 241–265. (doi:10.1017/jfm.2015.304)

[36] Blackburn HM, Sherwin SJ 2004 Formulation of a Galerkin spectral element—Fourier method for three-dimensional incompressible flows in cylindrical geometries. J. Comput. Phys. 197, 759–778. (doi:10.1016/j.jcp.2004.02.013)

[37] Mao X, Sherwin SJ, Blackburn HM 2011 Transient growth and bypass transition in stenotic flow with a physiological waveform. Theor. Comput. Fluid Dyn. 25, 31–42. (doi:10.1007/s00162-009-0167-9)

[38] Rist U, Augustin K 2006 Control of laminar separation bubbles using instability waves. AIAA J. 44, 2217–2223. (doi:10.2514/1.17518)

[39] Wilson J, Schatzmann D, Arad A, Seifert A, Shtendel T 2013 Suction and pulsed-blowing flow control applied to an axisymmetric body. AIAA J. 51, 2432–2446. (doi:10.2514/1.J052333)

[40] Poncet P, Hildebrand R, Cottet G, Koumoutsakos P 2008 Spatially distributed control for optimal drag reduction of the flow past a circular cylinder. J. Fluid Mech. 599, 111–120. (doi:10.1017/S0022112008000177)

[41] Shukla R, Arakeri J 2013 Minimum power consumption for drag reduction on a circular cylinder by tangential surface motion. J. Fluid Mech. 715, 597–641. (doi:10.1017/jfm.2012.537)

[42] Wei M, Freund J 2006 A noise-controlled free shear flow. J. Fluid Mech. 546, 123–152. (doi:10.1017/S0022112005007093)

